# “MoSpec”: A customized and integrated system for model development, verification and validation

**DOI:** 10.1371/journal.pone.0316401

**Published:** 2025-01-02

**Authors:** Marcello Pompa, Simona Panunzi, Alessandro Borri, Laura D’Orsi, Andrea De Gaetano

**Affiliations:** 1 Institute of Systems Analysis and Informatics “A. Ruberti” (IASI), National Research Council of Italy, Rome, Italy; 2 Institute for Biomedical Research and Innovation (IRIB), National Research Council of Italy, Palermo, Italy; 3 Dept. of Biomatics Óbuda University, Budapest, Hungary; Parul University Parul Institute of Technology, INDIA

## Abstract

**Background and objective:**

The growing availability of patient data from several clinical settings, fueled by advanced analysis systems and new diagnostics, presents a unique opportunity. These data can be used to understand disease progression and predict future outcomes. However, analysing this vast amount of data requires collaboration between physicians and experts from diverse fields like mathematics and engineering.

**Methods:**

Mathematical models play a crucial role in interpreting patient data and enable *in-silico* simulations for diagnosis and treatment. To facilitate the creation and sharing of such models, the CNR-IASI BioMatLab group developed the “Gemini” (MoSpec/Autocoder) system, a framework allowing researchers with basic mathematical knowledge to quickly and correctly translate biological problems into Ordinary Differential Equations models. The system facilitates the development and computation of mathematical models for the interpretation of medical and biological phenomena, also using data from the clinical setting or laboratory experiments for parameter estimation.

**Results:**

Gemini automatically generates code in multiple languages (C++, Matlab, R, and Julia) and automatically creates documentation, including code, figures, and visualizations.

**Conclusions:**

This user-friendly approach promotes model sharing and collaboration among researchers, besides vastly increasing group productivity.

## Introduction

Large volumes of data are these days collected and archived from patients in a variety of clinical settings. This phenomenon is the product of the evolution of analysis systems and the appearance of new diagnostic techniques [1–3]. The physician has thus in many cases observations of the clinical status of the patient at different points in time. With the advent of “omic” sciences, modern system of analysis and development of new computer languages, it is now possible to have a detailed picture of the patient condition at different stages of a possible disease. This abundance of clinical data represents an opportunity to satisfy two big needs: to understand the evolution of the disease, focusing on the causes, for diagnosis; and to provide a forecast of the evolution of the disease itself, for prognosis.

This rapid expansion of available data has determined the need for an interdisciplinary approach to their analysis, involving experts with different backgrounds (engineers, physicists, mathematicians, etc.).

The physician is thus no longer forced to reinterpret himself in order to conduct research in isolation, but can collaborate with other experts with emerging profiles, such as bio-mathematicians, bio-engineers, bio-informaticians.

In this context, the development of mathematical models describing patient pathophysiology plays an important and ever-expanding role in improving data interpretation for diagnosis, in optimizing existing protocols, in simulating scenarios for innovative medical treatments [3–12]. Modeling is at the basis of the possibility to conduct simulations, the so-called “*in-silico*” testing, which supplements biological “*in-vivo*” testing (*in-vitro*, *ex-vivo*, animal or patient experiments), abating costs and ethically reducing discomfort and suffering.

Implementing a mathematical model, however, requires the knowledge of a specific programming language and extensive coding. During this process, the risk of making errors, which involves spending time debugging and correcting the code, can be significant, depending on the complexity of the model and the level of expertise of the user. Software tools for simplifying mathematical model implementation and computation are described in the literature. Modelica [13], Simulink [14] and Comsol Multiphysics [15] are prominent examples for simulating physical systems. Modelica is an open-source platform from Linköping University and the Open Source Modelica Consortium. It provides a flexible framework for modeling diverse systems. Simulink is a commercial tool from MathWorks, offering a graphical environment for simulating dynamical systems. It includes a vast library of pre-built blocks and a user-friendly interface. Comsol Multiphysics is a commercial software excelling at coupling and solving multiphysics problems. It allows users to combine different physical phenomena for simulating complex real-world scenarios. Beyond these, Wolfram Alpha [16] is an online service that, while not a dedicated modeling tool, is a powerful computational knowledge engine aiding modeling tasks through its mathematical capabilities and the Wolfram Language.

In this context, the CNR-IASI BioMatLab group has developed a system, named “Gemini”, but ordinarily referred to as “MoSpec/Autocoder”, aimed at simplifying and speeding up mathematical model implementation. The system is designed for use by researchers with a basic knowledge of Ordinary Differential Equations and with the ability to translate into equations the problems at hand. While it has been developed in a biomedical context, and used to address mainly pathophysiological situations, the system is actually completely general and can be used in other domains without modifications. The motivation for the development and progressive improvement of the system stems from the need of a group of researchers, collaborating from several different geographical locations, at different levels of career progression and expertise, to synthetically and unequivocally sharing successive versions of the models they work upon, in an easy-to-understand format.

The system, consists of a combination of a so-called “MoSpec”, a customized Model Specification spreadsheet, with an Autocoder (Automatic code generation) program, a pre-compiled Matlab code taking the MoSpec in input. It is crucial to emphasize that “Gemini—MoSpec/Autocoder” is an independent and distinct tool from Google’s Gemini large language model. Our system is neither derived from nor aligned with the “Google—Gemini” model, it was not developed to achieve the same objectives, and our choice of the name Gemini actually pre-dates the appearance of the Google product of the same name.

Unlike the commercial nature of most of the tools mentioned above, the MoSpec/Autocoder is a free system that does not require specific programming knowledge but only basic spreadsheet knowledge for compilation, though accuracy is essential.

The system “Gemini—MoSpec/Autocoder” allows the researcher to completely specify mixed Algebraic and Ordinary Differential Equations models in the MoSpec, then run the Autocoder on it, obtaining in output working simulation code in C++, Matlab, and R (a Julia output implementation is also under way). This multi-language model implementation originated the name “Gemini” (“twins”). The Autocoder outputs complete LaTeX code for model description, including tables and integrating supplied figures, which can be used as a self-standing (“Companion”) report, or integrated into manuscripts intended for publication. The Autocoder also outputs compilable code for a so called “Visualizer”, a model implementation with a Matlab GUI and a C++ computing engine used to quickly visualize the effect of parameter changes on the time-course of the state variables and produce graphs thereof. Finally, the Autocoder provides compilable code that can be directly inserted into a standard PHP Web-server implementation, to offer Machine-to-Machine (M2M) access to the model via a SOAP protocol.

The work outlines the procedure to upload any mathematical model, described according to the MoSpec specifications, onto the Gemini system to provide users with a MATLAB, R and Julia implementation of the model itself.

## Theoretical framework

The present section provides a brief overview of the programming languages connected with the Gemini system, together with a brief discussion of the Verification and Validation procedures, which are of fundamental importance for asserting that the model is able to represent the phenomenon of interest.

### Verification and validation

Verification and validation (V & V) are two distinct procedures used to check that certain systems, services or, more in general, products fulfill their intended purpose and satisfy requirements and specifications [17]. In our context the goal is that of assuring that a software model implementation correctly reproduces numerically the behavior of the pathophysiological system under investigation.

- **Verification**: may be defined as the process of assuring that a product, service, or system meets the needs or requirements of the users. In our context this means that the software implementation of a model works as specified in the mathematical description of the model itself, without bugs.
- **Validation**: is the process of evaluating whether or not a product, service, or system complies with regulations, requirements, specifications, or imposed conditions. It is typically performed by testing the system with real users to see if it meets their needs. Again, in our context this means that the implemented model must adhere to experimental observations, both those which are already available and those which are to be obtained in the quest for model falsification according to Popper [18].

Boehm [19] characterised the difference between the two procedures in the following two statements:

1. Verification is building the system right.
2. Validation is building the right system.

According to [20] the final aims of the V & V process are therefore:

1. to detect bugs in the implementation and resolve them;
2. to detect problems or highlight important missing parts in the application.

Similarly, the U.S. Food and Drug Administration (FDA) guidelines report the following definition for validation and verification [21], respectively:

- “Software verification provides objective evidence … for consistency, completeness, and correctness of the software and its supporting documentation … and provides support for a subsequent conclusion that software is validated”.
- “[validation entails] confirmation by examination and provision of objective evidence that software specifications conform to user needs and intended uses, and that the particular requirements implemented through software can be consistently fulfilled.” (in our context, that the model behaves according to physiology)

Notice in the FDA’s definition of verification the emphasis placed on documentation: this is important to guarantee that developers and users understand and agree on what is being implemented. Appropriate, exhaustive and precise documentation makes it simpler to improve on the current model to address more complex issues or different application areas.

It is evident that V & V of (biomedical) model construction is a complex task that requires a variety of skills and techniques. As such, it is typically carried out by a multidisciplinary team of professionals, including mathematicians/statisticians, engineers, physiologists. The proposed *formalized system* is designed to support the Verification process. It proves to be a particularly useful tool in a development environment to allow collaborators, working on the same project, to communicate and to understand each other’s contribution to the development. It could be imagined as a universal language for the work group, a structured method to develop new projects. The use of such a *formalized system* allows to perform the verification process in a standardized, predictable, robust way: if each new project is tackled in the same way, following the same instructions, and the same steps, then making substantial errors becomes more difficult. In other words, a *formalized system* leads the developers to follow strict rules in the development phase: due to its very nature, a standard operating procedure makes development less liable to possible implementation errors. Furthermore, error detection by co-workers becomes easier as the common standards and formats adopted simplify communication and collaboration. Specifically, the *formalized system* presented in this paper (named “MoSpec” and comprehensively outlined in the following subsections) was specifically designed to streamline and enhance the Verification process during the development and programming implementation of a mathematical model.

Besides facilitating verification through the use of standardized fields and automatic checks, the system specifically provides for a verification step by producing, in the “companion” LaTeX document, side-by-side expressions for each equation’s right-hand-side (one in mathematical notation, one in computational notation): it thus becomes easier for the modeler to check that the (automatically produced) code captures exactly what the originally imagined equation was meant to express. In addition, the “Visualizer” tool, presented in the “Visualizer and C++ Implementation” subsection, also facilitates the Validation process, enabling users to employ experimental data to calibrate the parameters of a mathematical model and to assess whether the specified model is able to reproduce the expected behaviour.

### Matlab, R, Julia, C++, HTML and PHP

After the theoretical construction of a mathematical model, which represents the phenomenon under study with a certain level of simplification, it is necessary to implement the model equations in a programming language to obtain simulations regarding the system behavior. In the following a brief description of the most commonly used programming languages is provided:

- **Matlab** is a proprietary programming language by MathWorks [22]. It is an interactive environment for handling variables, calculations, and data; its coding logic resembles C++, making it easier to convert MATLAB code to C++.
- **C++** is a high-level language extending C, often compiled for systems and embedded software [23]. It’s known for its performance, efficiency, and flexibility.
- **R** is a free, high-level language for statistics and graphics [24]. It’s easy to learn and powerful for tasks like data analysis, visualization, and modeling. R is highly customizable with a vast community of contributors.
- **Julia** [25] is a new, high-level language combining C/C++ speed with Python/R ease. It’s great for numerical analysis and computational science. Julia is open-source, actively developed within a strong community.
- **HTML** is the foundation of web pages. It uses tags to define a document’s structure and content for web browsers. These tags tell the browser how to display elements like headings, paragraphs, images, and more. [26].
- **PHP** is a scripting language for web development [27]. It runs on web servers, producing HTML, images, and more. PHP can perform system tasks like file creation and manipulation, making it efficient and flexible.

## The BioMatLab “Gemini” (MoSpec/Autocoder) system

**Fig 1:**
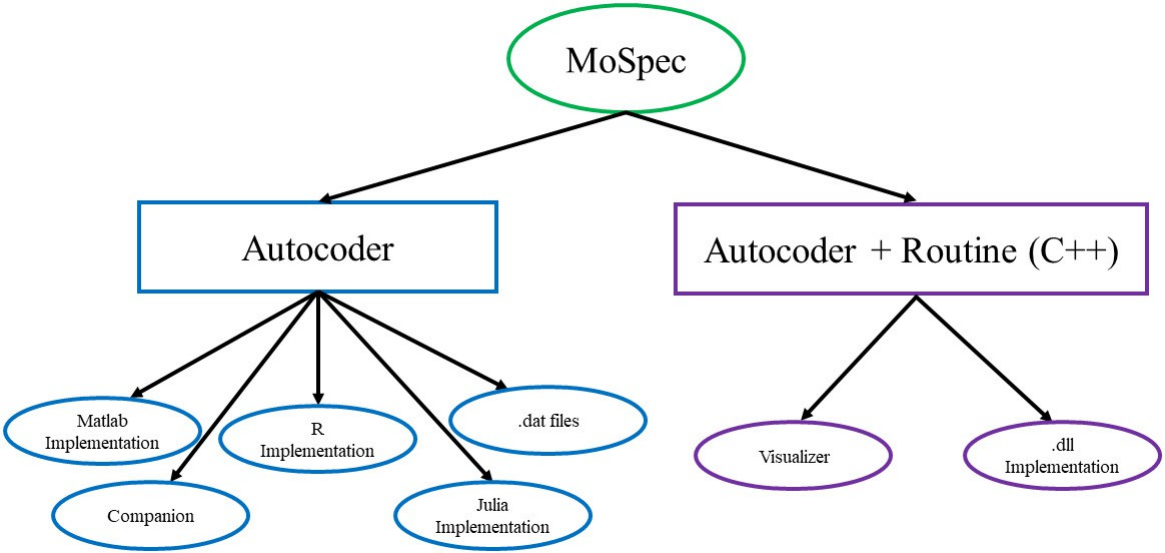
Block diagram of the MoSpec/Autocoder functioning.

The MoSpec, is an OpenDocument Spreadsheet template developed at the CNR-IASI BioMatLab, implementing internal standard content for addressing the issues and needs presented in the “Theoretical framework” section. The MoSpec is the formalized document, which completely describes in pseudo code a mathematical model, and which is used in pair with the Autocoder, a BioMatLab proprietary Matlab script: the Autocoder takes in input the MoSpec and automatically produces the implementation of the mathematical model described there in different programming languages (Matlab, C++, R and Julia). The information included in the MoSpec (such as parameter and variable descriptions as well as the relative units of measurement) also allows the Autocoder to generate a complete LaTeX document, the “Companion”, which reports the model equations, pairing their mathematical form with their “verbatim” computational definition, along with tables for all model variables and parameters. The system also allows the integration, in the final LaTeX document, of figures and diagrams as referred to in the MoSpec. The diagram block in Fig 1 shows the output of the MoSpec/Autocoder system.

### MoSpec

The MoSpec (Model Specification spreadsheet) is the place where the modeler specifies all information regarding the model: so much so that at the CNR IASI BioMatLab a given “model” is intended to be uniquely determined by the corresponding MoSpec, which is named according to the model type or application (the name), the model itself (version), the implementation (sub-version) and the edition of the describing MoSpec (sub-sub-version). So for instance, if in the class of models representing romantic love between people, model 2 contemplates two lovers exhibiting certain nonlinear dynamics, it has been iteratively corrected (hence with different computed results) four times and the corresponding MoSpec has been embellished and completed three times, the current version of the MoSpec is named “RomanticLove.MoSpec V02.04.03”.

As mentioned above, the MoSpec is a “.ods” file (we use the free program LibreOffice [28], organized in 11 obligatory sheets, with additional “instructions” and “notes” sheets, and with zero or more “support” sheets helping the modeler to handle parameter values or conversions, or in general incorporating useful material. In the obligatory sheets, the cells which must be filled by the modeler have a yellow background: all other cells are reserved for system maintenance. The spreadsheet must be compiled in its entirety.

**Fig 2:**
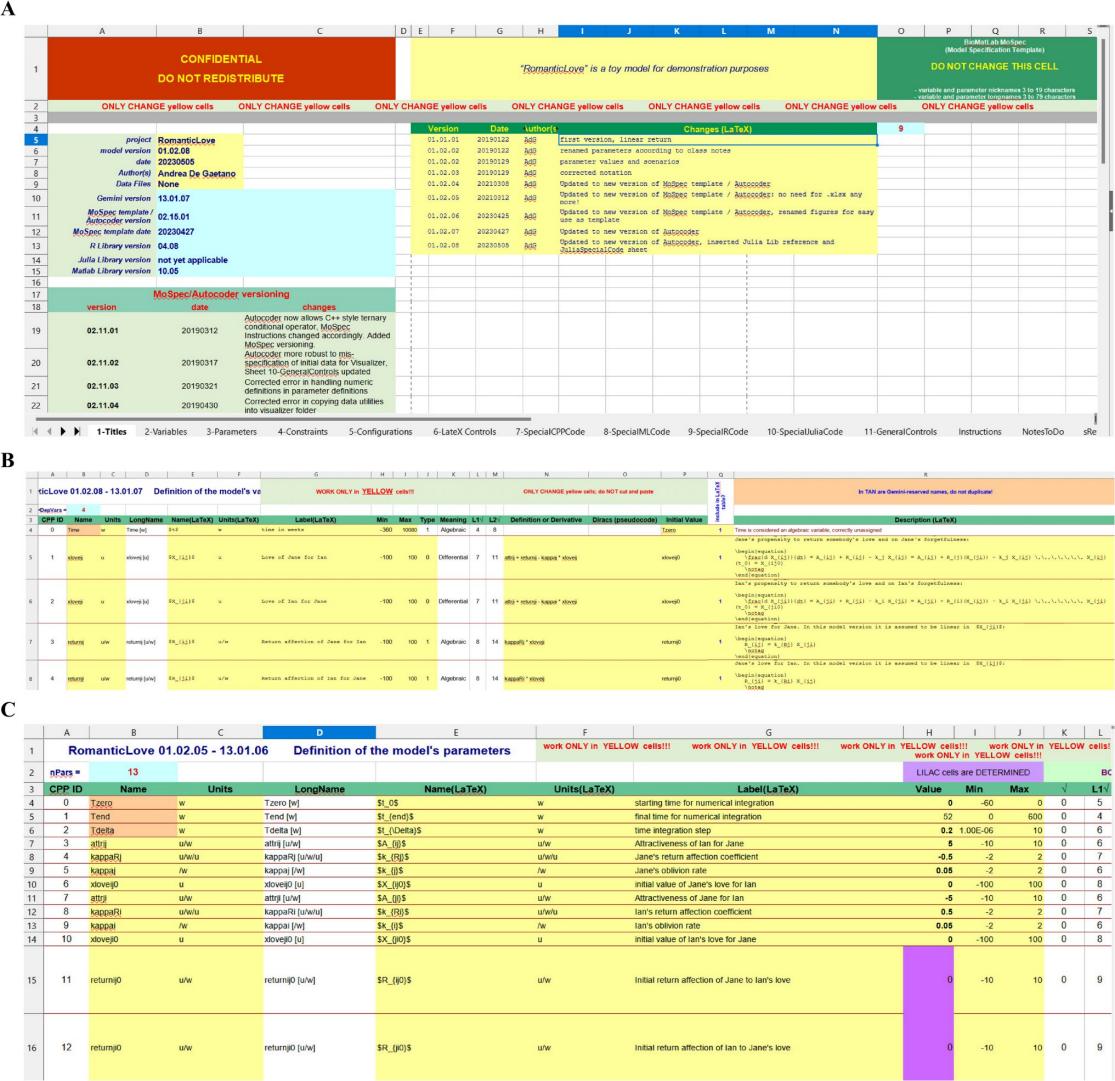
The main MoSpec sheets. (A) “1-Title” is the sheet reporting the general information of the current project; (B) “2-Variable” is the sheet including variable description; (C) “3-Parameters” is the sheet describing model parameters.

The first sheet (“Titles”) of the MoSpec contains general information about the current project (Fig 2, panel A): name, dates, versions and changelog of any modification made during the project development.

The second (“Variables”) sheet contains all the information regarding the state variables (Fig 2, panel B) including name, unit of measurement, description, range of variation, pseudo code defining the variable (either algebraically or differentially), initial conditions and the LaTeX code for the Companion.

The third sheet (“Parameters”) has approximately the same structure of the “Variables” sheet and contains a description of the model parameters (Fig 2, panel C) accompanied by their reference value, admissible minima and maxima, etc. The two terms “free” or “determined” are here used to indicate those (“determined”) parameters that are computed as function of other (“free”). To make the distinction between the two different types of parameters more immediately visible to the user the cells correspondent to the “determined” parameters are colored purple.

**Fig 3:**
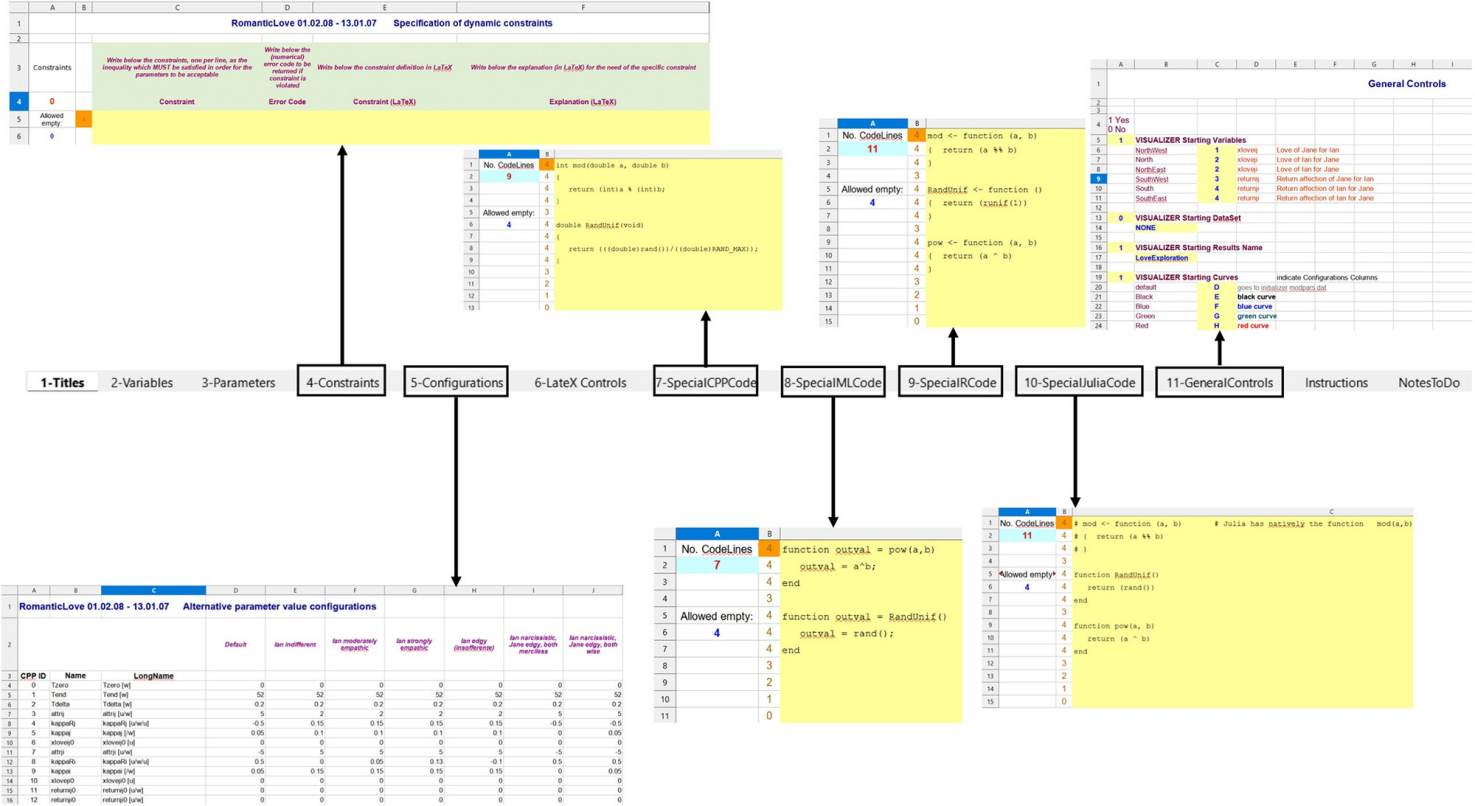
The MoSpec sheets.

The fourth sheet “Constraints” (Fig 3) collects restrictions (if any) on the model parameters, one per line, which must be satisfied during the estimation or calibration procedure to obtain a coherent model prediction.

The sheet allows to declare, for each parameter constraint, the error code (a number) to be shown in case the constraint is not satisfied, as well as corresponding LaTeX description of the constraint.

The fifth sheet “Configurations” (Fig 3) collects possible model parameter vector configurations to allow different scenarios (e.g. the “Default” scenario or the “Ian indifferent” scenario of the RomanticLove model in Fig 3).

**Fig 4:**
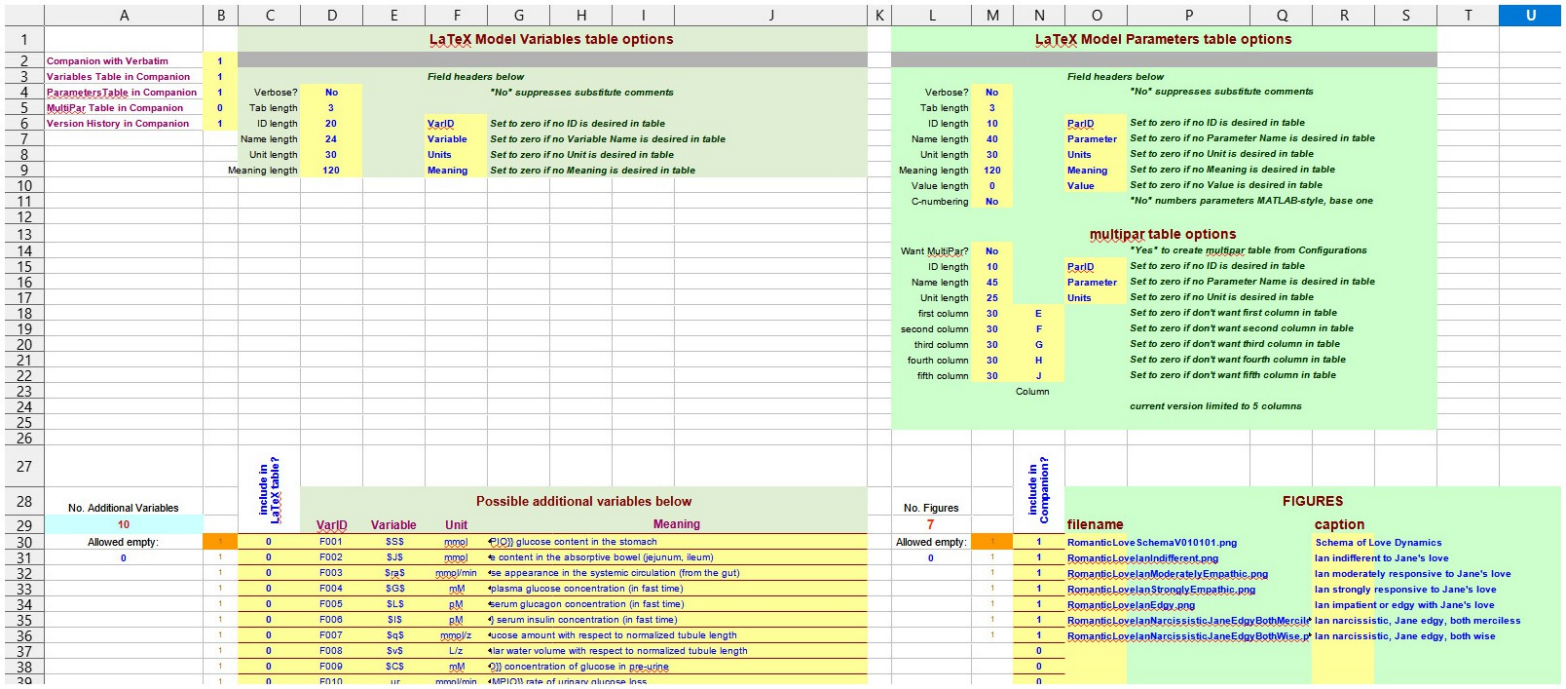
“LaTeX Controls” sheet.

The sixth sheet “LaTeX Controls” (Fig 4) collects a series of commands useful to modify tables (as for example the length of the table cells: “Tab length”, “Name length”, etc.) and figures (if any) for the generation of the “Companion” LaTeX file.

While the tables are automatically generated from the information contained in the “Variables” and “Parameters” sheets, the figures are included in the LaTeX file only if provided and their names indicated in the present sheet.

The seventh (“SpecialCPPCode”), eighth (“SpecialMLCode”), ninth (“SpecialRCode”) and tenth (“SpecialJuliaCode”) sheets (Fig 3) are used to declare in C++ code (sheet 7), in Matlab code (sheet 8), in R coe (sheet 9) and in Julia code (sheet 10) some specific functions useful for the model integration. In particular, since some frequently used functions are differently indicated in the four languages, an alternative, common form is used in the variable and parameter definition pseudocode and this form is then defined for the four languages in their own syntax: as an example, the pseudocode “RandUnif()” function translates into the R “runif()” or the Matlab “Rand()” functions.

Sheet 11 “GeneralControls” (Fig 3) allows to define a series of options for the Visualizer tool, such as the model variables to be shown at the opening of the Visualizer (the number of whose graphical windows is limited), or the data sets to be loaded by default.

The MoSpec “Instructions” sheet is merely a collection of instructions for the correct compilation of the MoSpec itself, whereas the “NotesToDo” sheet includes communications to be shared among the users working on the project.

**Fig 5:**
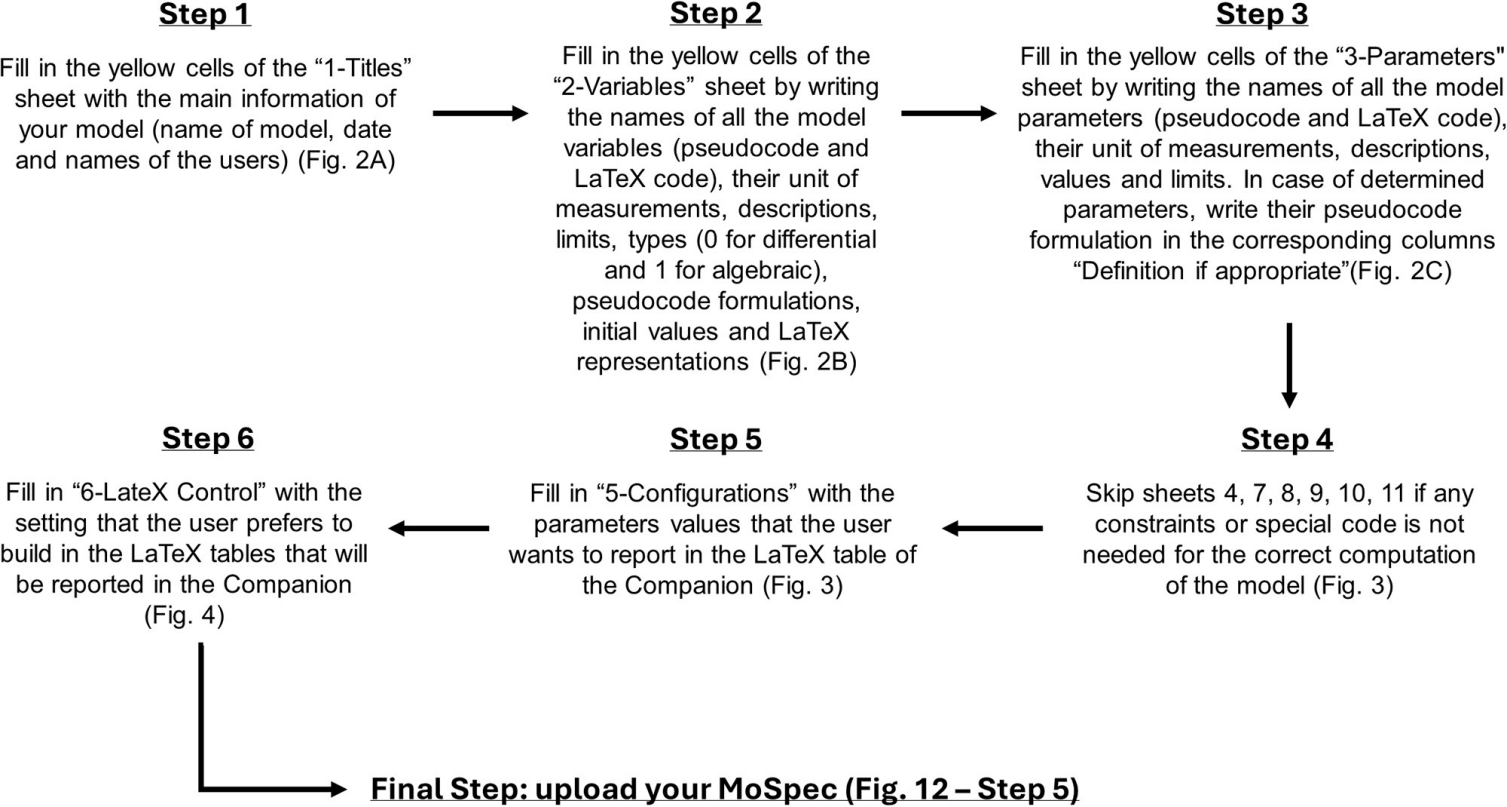
Workflow for MoSpec sheet compilation and upload.

Fig 5 illustrates the workflow for correctly compiling the MoSpec sheets.

While the “.ods” MoSpec file is considered the single valid model specification, the Autocoder actually supports both “.ods” and “.xlsx” file formats.

### Autocoder

**Fig 6:**
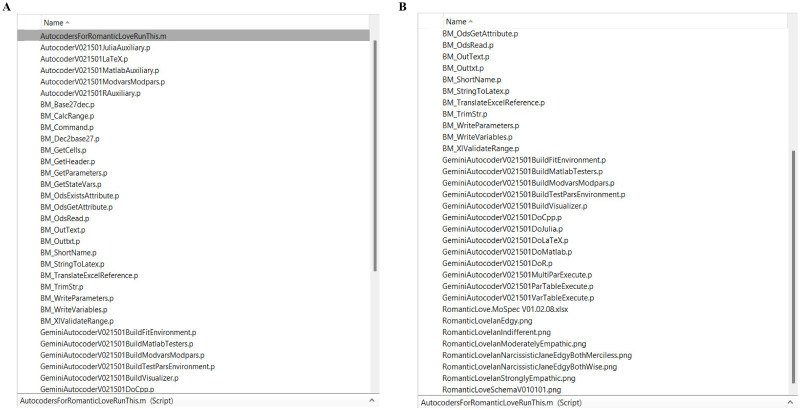
(A-B) Autocoder Matlab files generating model implementation in Matlab, R and Julia programming languages and producing the LaTeX companion file.

The Autocoder is a Matlab program written at the BioMatLab for the interpretation of the information provided in the MoSpec (Fig 6).

While the input into the Autocoder is a valid MoSpec file, its output is a series of files in the diverse programming language for the simulation and visualization of the model described in the MoSpec.

#### Matlab, R and Julia

**Fig 7:**
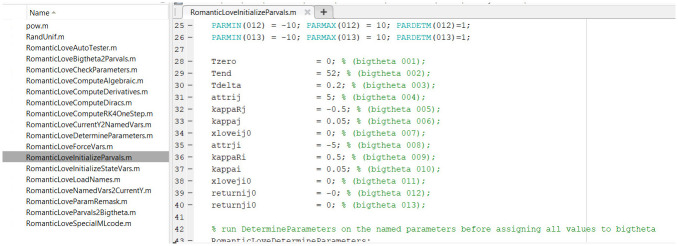
Matlab model implementation files produced by the Autocoder.

**Fig 8:**
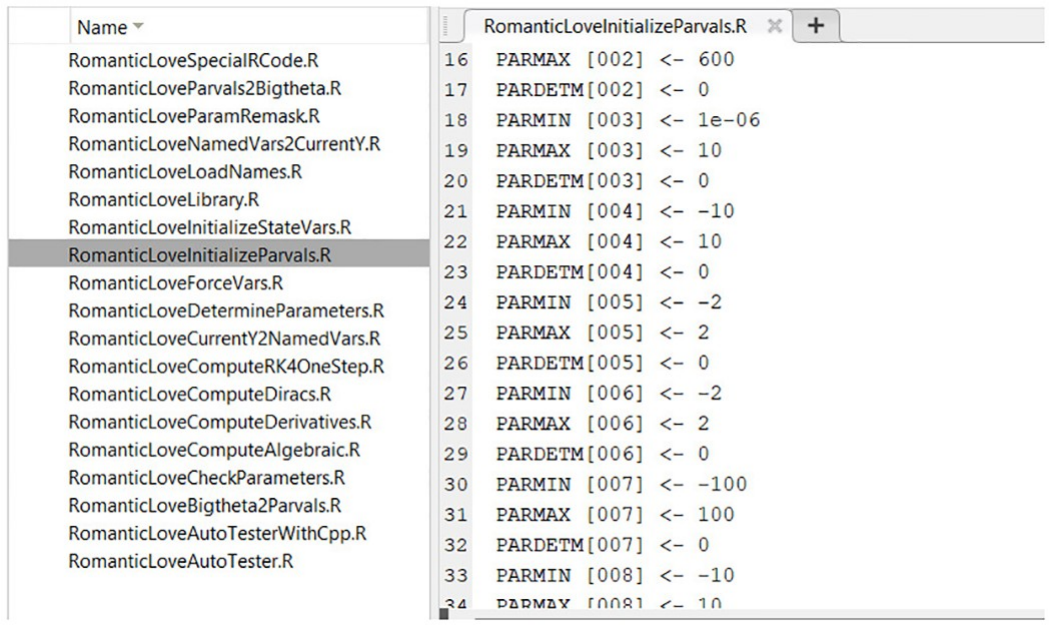
R model implementation files produced by the Autocoder.

**Fig 9:**
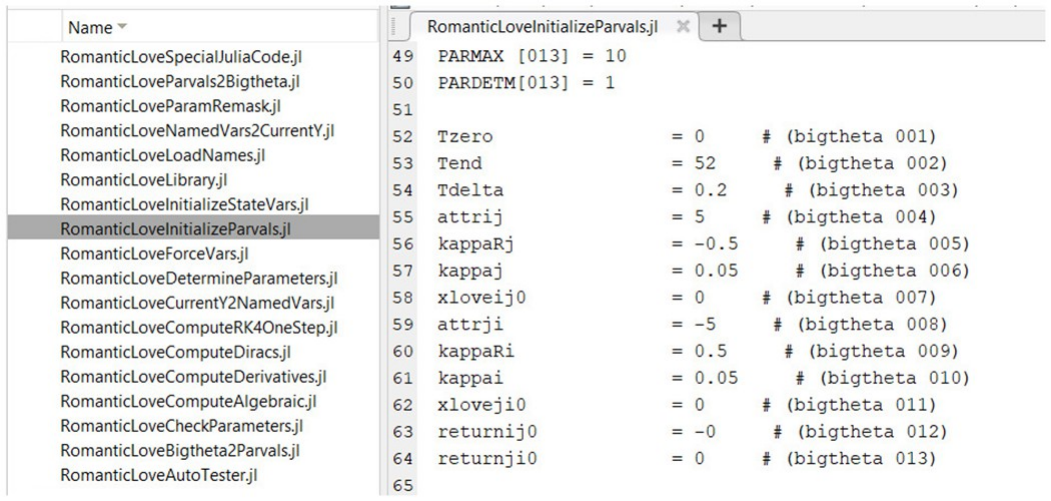
Julia model implementation files produced by the Autocoder.

The execution of the Autocoder (which is itself a Matlab program) creates and populates separate folders containing all the necessary routines for the numerical integration of the model in each of the three languages (Matlab, R and Julia). The routines have the same names across languages and refer to similarly-named variables, to make switching context and debugging simpler. Files in Matlab, R and Julia code are shown in Figs 7–9, respectively.

For example, the Matlab implementation of the model is executed by running the “*ProjectName*AutoTester.m” file. The output of the Matlab code is generated in terms of plots of the model variable time courses. The numerical integration method used by the system is the fourth order, fixed-step Runge-Kutta.

#### C++

The Autocoder also produces model-specific source C++ code implementing the numerical integration of the model and referring to a BioMatLab library of standard mathematical functions (e.g Runge-Kutta integration, global optimization, datafile access,…) for eventual compilation within a Microsoft Visual Code C++ Community Edition 2021 environment. Such compiled C++ code takes two forms:

- it produces a self-standing “.exe” file callable from a Windows environment with a string specifying all necessary parameter values, returning a string containing all model predictions at the desired times; this “.exe” implementation of the model is the fast computational engine used to deliver model predictions via machine-to-machine PHP SOAP interface or through a human user interface (see below “Online Platform”). The Companion, in pdf format, can also be downloaded from the online platform.
- it produces the necessary compiled Dynamic Link Libraries (“.dll”) for the numerical integration of the model within the Visualizer model validation tool, see below.

#### Visualizer

**Fig 10:**
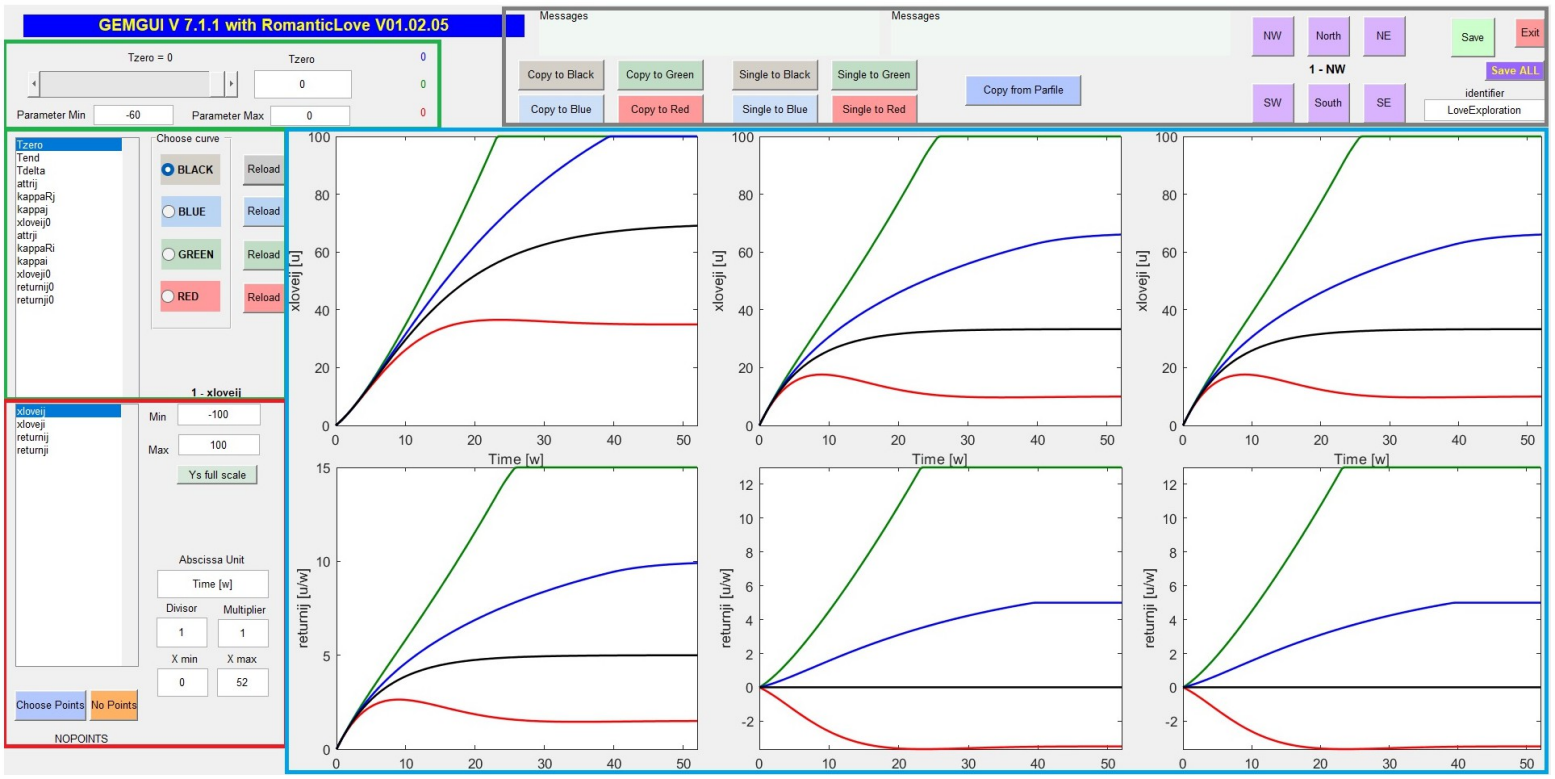
Visualizer interface: Green countered boxes report the model parameters and their control bar for values modification. The chosen configuration (black, blue, green and red) correspond to different parameter values resulting in different (corresponding) model predictions in the adjacent plots; red countered box shows the model variables along with their minimum and maximum allowed. The button “Choose Points”, at the bottom of the box, loads external data to be superimposed to the model predictions. Buttons in the grey countered box enable the choice of the quadrant (NW, North, NE, etc.) where to plot the different model variables (light-blue countered box). This grey box contains also buttons for copying a value of a parameter on a specific parameter vector configuration (black, blue, green or red).

The Autocoder produces the Matlab code supporting a graphical user interface for model prediction visualization: the “Visualizer” (Fig 10), as well as the (.csv format) supporting parameter and variable files (containing default and scenario values, acceptable limits and so on).

The interface is composed of different zones: the parameter section, which allows to modify the parameter values (defaults being automatically extracted from the MoSpec); the variable section, which allows to choose the state variables to visualize in the adjacent plot section; the control section, which presents different output options, one of which the possibility to add and visualize observed data (related to one or more state variables). This allows to visually calibrate the model parameters to explore different scenarios, or before an optimization procedure for parameter estimation is performed.

In this implementation, Matlab only deals with the interface allowing the user to change model parameters or specifying datasets, as well as returning forecast graphics. The model integration is however performed in .dll’s obtained from compiling the C++ sourcecode generated by the Autocoder, which makes model integration very fast: for example, by keeping pressed the interface button corresponding to some parameter increment, the time-course graphs of the state variables are seen to move rapidly to reflect successive values of the parameter as it varies in its range of definition.

The Visualizer environment represents a robust tool to check the behavior of the model, with respect to all state variables, depending on the parameter choices: this is extremely useful both when first assessing the likely range of values of previously unknown parameters and when validating the model behavior with respect to plausible parameter values.

#### The LaTeX “Companion”

**Fig 11:**
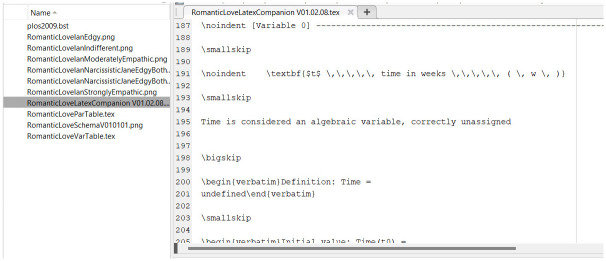
LaTeX model description files produced by the Autocoder.

A series of LaTeX files (Fig 11) are also provided as output of the Autocoder: these files are used to generate the Companion, the document containing the mathematical equations, parameter and variable descriptions.

Furthermore, two “.dat” files (“*ProjectName*.modpars.dat” and “*ProjectName*.vars.dat”) are also produced by the Autocoder. These files contain a structural representation of all the information related to the model parameters and variables and are exploited by the C++ implementation, the Visualizer and the online platform.

#### Online platform (HTML, PHP)

At CNR-IASI we have developed a standard HTML user interface and a PHP platform resident on our servers, able to accept and satisfy requests for service coming from the Internet.

Any model, successfully implemented, verified and validated, can afterwards be loaded on the CNR server, so that it becomes usable in user-to-machine and machine-to-machine modes. In user-to-machine mode the user can insert or modify the model’s parameter values from an HTML interface and see on the same in the same interface page the corresponding graphical representation of the model forecasts of the time course of the state variables. machine-to-machine mode the user’s softare can generate a SOAP call to the server, containing the simulation parameters and the desired evaluation time-points, and the server returns to the calling software the corresponding model forecasts at the desired time-points.

The platform can be found at the address:

http://biomatlab.iasi.cnr.it/models/login.php

The following is a list of the models currently provided:

- Corona: a multinode generalized Susceptible—Exposed—Infectious—Removed (SEIR) model incorporating major features of the COVID19 pandemic in Italy;
- Guyton: a celebrated maximal model of the vascular system [29];
- OGTToy: a minimal model for the representation of data from an Oral Glucose Tolerance Test (OGTT);
- RomanticLove: a simple model that represents the dynamics of attraction between two people, similar to the Rinaldi romantic relationship models [30, 31];
- Sorensen: a complex, maximal model of the human glucose-insulin system for both normal and diabetic subjects (our implementation from the original Sorensen’s PhD dissertation and subsequent corrections) [32];
- UVaPadova: another maximal model of the human glucose-insulin system for both normal and diabetic subjects (or implementation from published equations) [33];
- Dodeg: a simple, realistic mathematical description of cardiovascular dynamics after hemorrhagic shock [34];
- Zenker: a 2007 model portraying the evolution over time after blood loss of clinically relevant variables, such as the mean arterial pressure (MAP), the heart rate (HR), the cardiac output (CO), and the central venous pressure (CVP) [35];
- ZenCur: an upgraded version of the 2007 Zenker model for the representation of the hemorrhagic shock [36].

**Fig 12:**
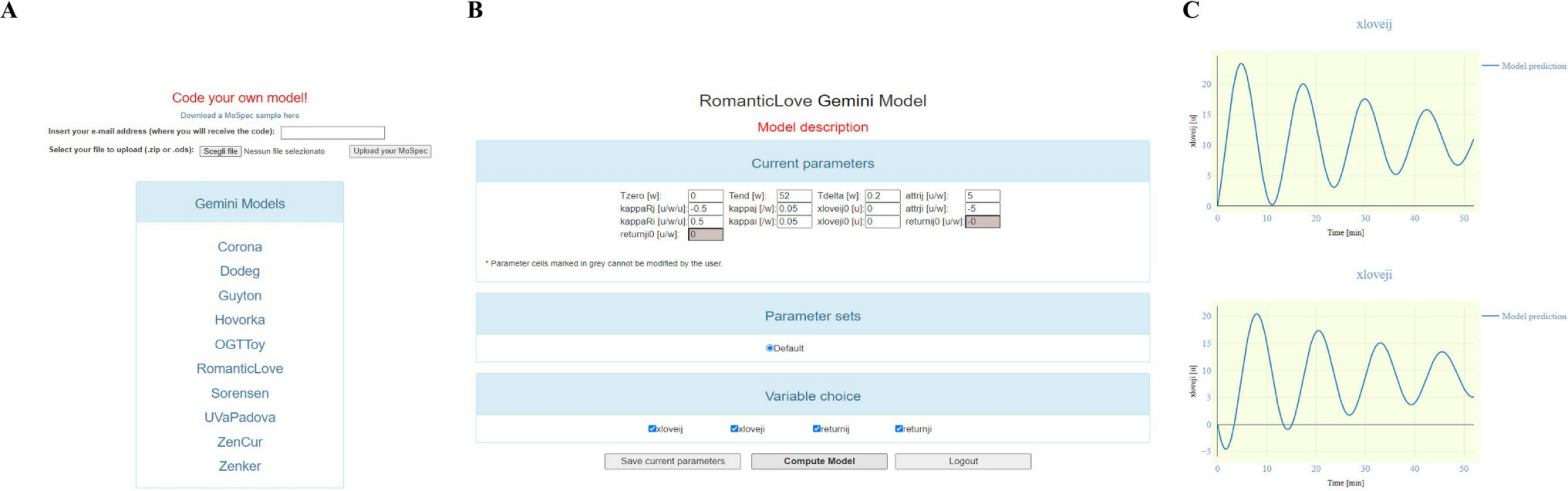
Screenshots of the online platform. (A) webpage reporting the available models provided by the BioMatlab server; (B) webpage related to the RomanicLove model and showing the list of model parameters and variables, as well as the buttons for model simulation; (C) plots of two of the RomanticLove model variables.

Any user interested in simulating one of these models can access the platform as “Guest”. Following access, a webpage reporting the complete list of the models (Fig 12, panel A) will appear.

After having selected the desired model (for example the RomanticLove model), the user will be sent to the model-specific page showing the lists of model parameters and variables, along with their units of measurement and reference or starting values, respectively (Fig 12, panel B). The user has the possibility to change any parameter value (if the parameter is not determined, see above) and to obtain the corresponding model predictions by clicking the “Compute Model” button (Fig 12, panel C).

After logging into the platform as a guest, it is also possible to download a MoSpec sample (at the “Download a MoSpec sample here” link at the top of Fig 12). The file can be modified to include structured information about a specific model that the user wants to implement. It can be a model from literature or their own model. The procedure to follow is summarized in the following steps:

- once opportunely filled out with all the necessary information, the “.ods” file must be renamed with the name of the current project (chosen by the user), and saved in “.ods”;
- the file can be uploaded as a single “.ods” file or as a “.zip” file if the user wants to include figures into the Companion that will be generated;
- once the file has been uploaded the user will receive a notification that the MATLAB, R and Julia implementation of the model, as well as the model LaTeX description, will be available via email (which must be provided in the “Insert your e-mail address” text box, Fig 12) within 2 working days.

**Fig 13:**
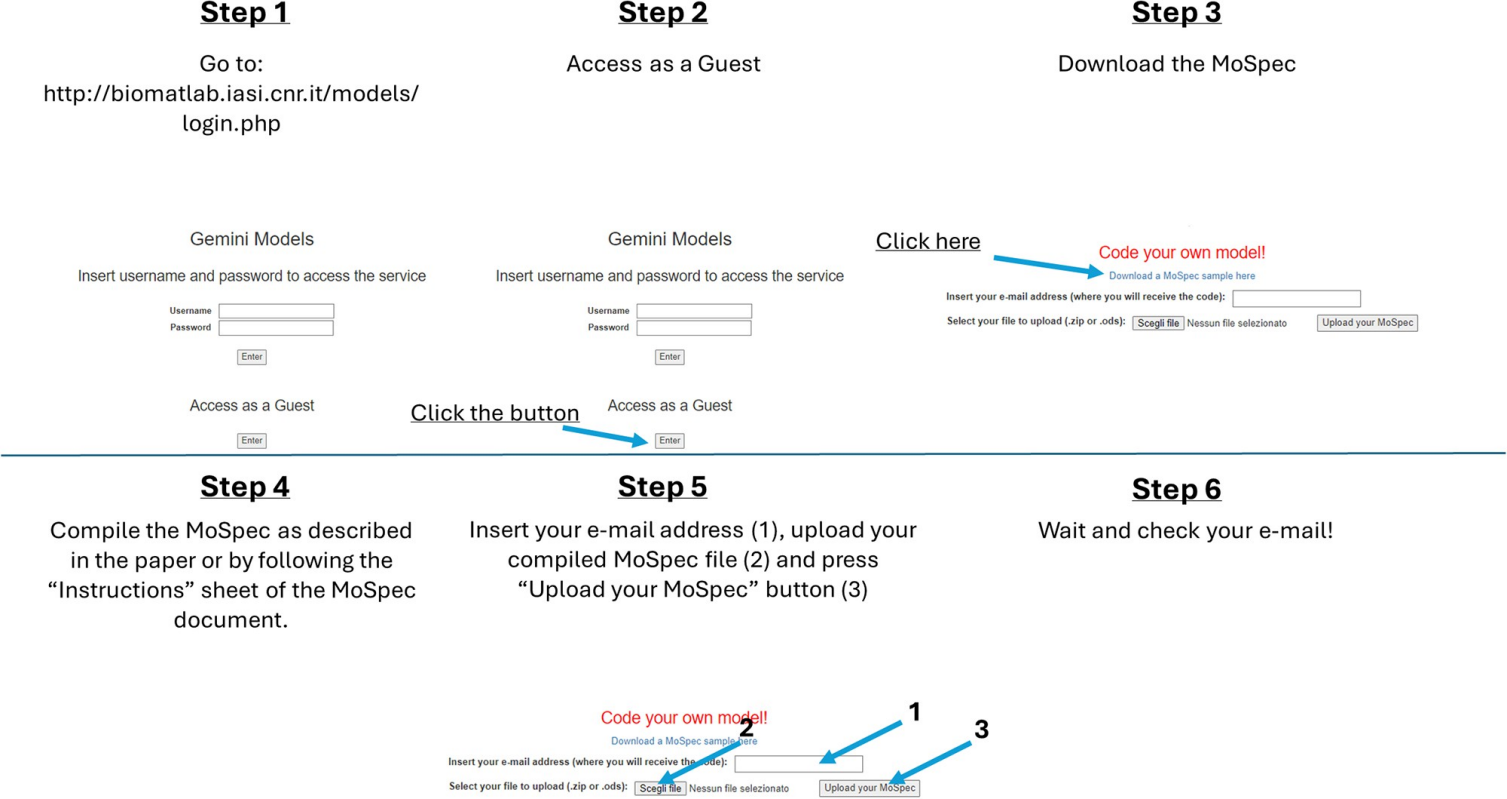
Step by step procedure for Gemini online platform access and for MoSpec download and upload.

Fig 13 summarizes the above steps.

## Discussion

Mathematical models, applied across various medical fields, from basic research to clinical practice, play a crucial role in modern medicine, enabling researchers to better understand, diagnose, treat, and prevent diseases. They are employed to simulate the behavior of different biological systems and can be useful instruments for physicians to make informed decisions regarding diagnosis and prognosis. Mathematical models may also be of great help in optimizing medical treatments by determining the most effective dosage of a drug or in designing novel therapeutic approaches. Furthermore, their ability in providing insights into the underlying mechanisms of pathological conditions appears to be of great importance in understanding disease progression, identifying therapeutic targets, and developing personalized medicine strategies.

Since mathematical models are becoming increasingly sophisticated and accurate due to technological advancements, it is clearly very useful to have available such tools that simplify and accelerate the correct implementation of standard mathematical models.

With the aim of addressing this objective and of rendering the modelling approach more feasible and accessible, the BioMatLab group has developed a system, easy to use, which is the result of a synergistic interaction between different programming languages and a customized spreadsheet, the MoSpec (Model Specification) file. Starting from the spreadsheet template containing different ad-hoc formatted sheets, the system allows automatic code generation for model implementation in different programming languages.

While other tools are available for implementing mathematical models, our system provides a free solution requiring no specific programming knowledge. Users only need to fill out a spreadsheet defining the model equations. This allows even non-programmers to produce and test ODE model implementations, which are automatically verified. Unlike other tools, our system also offers an informative document including tables, figures, and equations for easier scientific paper drafting.

It is to be stressed here that in our daily activity in the support of biomedical investigations our group also develops other kinds of (more mathematically sophisticated) models, based on Stochastic Differential Equations [37], Fractional Differential Equations [38, 39], Delay Differential Equations [40], Partial Differential Equations [41], even Fractional Stochastic Differential Equations [42]: such kinds of models are not easily handled by the current version of the MoSpec/Autocoder system. However, the bread-and-butter of everyday modeling activity still concerns Nonlinear Ordinary Differential Equations (ODE) [6, 9, 32–34, 36, 43–47], and for these the MoSpec represents a huge improvement in rapidity and reliability of implementation. It is also to be stressed that in a multi-disciplinary group not everybody is familiar with all details of coding for ODE models: even relatively junior figures can quickly become effective in contributing to the group’s production by using the MoSpec.

It should be noticed that in a multidisciplinary group different professionals tend to prefer different computing languages: R for our statisticians, Matlab for our engineers, C++ for our IT and Julia for those of us more attuned to recent developments: the system allows each of these users to work with their favorite language. The automatically generated LaTeX companion in particular provides equation-by-equation comparison of the mathematical formulation (decided upon at model design phase) with the corresponding computing code, facilitating verification. It also provides the source-code for tables of variable and parameters to be directly incorporated into manuscripts for publication.

By requiring the user to routinely follow specific steps and clear instructions, thus allowing a standardized check for errors, the proposed system has proved to be an extremely useful tool for following all the Verification and Validation phases necessary for the exact implementation of our models. Furthermore, the MoSpec has become a shared language for people working in a group, structuring the development of any new project: the common standards and format simplify and improve communication and the gain in development speed increases the number of new projects that can be simultaneously undertaken.

A first challenge associated with the use of the MoSpec system is the learning curve involved in accurately compiling the necessary spreadsheets. While this may initially require practice, proficient use can quickly be achieved. Although the MoSpec compilation process offers limited flexibility, this constraint actually contributes to its strengths: by requiring users to follow a standardized format, the system ensures implementation consistency and facilitates verification. While this approach may restrict user creativity, it also minimizes the risk of errors commonly encountered in less structured systems. Also, standardization is extremely useful in a group work environment, making it possible for different people to be brought rapidly up-to-date on unfamiliar projects. It’s important to note that the Autocoder outputs remain fully editable, providing users with opportunities for customization and tailored code script generation.

In conclusion, the CNR-IASI BioMatLab “Gemini MoSpec/Autocoder” system, which has become the standard procedure of our group for ODE model development, appears to be an extremely useful tool for the implementation, verification and validation of mathematical models.
